# Causal effects of gut microbiota on the prognosis of ischemic stroke: evidence from a bidirectional two-sample Mendelian randomization study

**DOI:** 10.3389/fmicb.2024.1346371

**Published:** 2024-04-08

**Authors:** Anning Zhu, Peng Li, Yuzhou Chu, Xiuxiang Wei, Jiangna Zhao, Longfei Luo, Tao Zhang, Juntao Yan

**Affiliations:** Department of Tuina, Yueyang Hospital of Integrated Traditional Chinese and Western Medicine, Shanghai University of Traditional Chinese Medicine, Shanghai, China

**Keywords:** gut microbiota, ischemic stroke, functional outcome, causal relationship, Mendelian randomization

## Abstract

**Background:**

Increasing research has implicated the possible effect of gut microbiota (GM) on the prognosis of ischemic stroke (IS). However, the precise causal relationship between GM and functional outcomes after IS remains unestablished.

**Methods:**

Data on 211 GM taxa from the MiBioGen consortium and data on prognosis of IS from the Genetics of Ischemic Stroke Functional Outcome (GISCOME) network were utilized as summary-level data of exposure and outcome. Four kinds of Mendelian randomization (MR) methods were carried out to ascertain the causal effect of GM on functional outcomes following IS. A reverse MR analysis was performed on the positive taxa identified in the forward MR analysis to determine the direction of causation. In addition, we conducted a comparative MR analysis without adjusting the baseline National Institute of Health Stroke Scale (NIHSS) of post-stroke functional outcomes to enhance confidence of the results obtained in the main analysis.

**Results:**

Four taxa were identified to be related to stroke prognosis in both main and comparative analyses. Specifically, genus *Ruminococcaceae UCG005* and the *Eubacterium oxidoreducens* group showed significantly negative effects on stroke prognosis, while the genus *Lachnospiraceae NK4A136* group and *Lachnospiraceae UCG004* showed protective effects against stroke prognosis. The reverse MR analysis did not support a causal role of stroke prognosis in GM. No evidence of heterogeneity, horizontal pleiotropy, and outliers was found.

**Conclusion:**

This MR study provided evidence that genetically predicted GM had a causal link with post-stroke outcomes. Specific gut microbiota taxa associated with IS prognosis were identified, which may be helpful to clarify the pathogenesis of ischemic stroke and making treatment strategies.

## Introduction

Stroke remains a severe health problem that causes death and long-term disability worldwide, resulting in increased economic and social burden (Herpich and Rincon, 2020). More importantly, such negative impact of stroke will increase as people age across the globe (Murray and Lopez, 2013). Stroke is usually classified into two types: ischemic and hemorrhagic. Ischemic stroke (IS) is the most prevalent one, accounting for approximately 70%−85% of all stroke cases globally (Pluta et al., 2021; DeLong et al., 2022). At present, effective treatments targeting IS, such as thrombolytic therapy (Li et al., 2021; Zhao et al., 2022), thrombectomy (Winkelmeier et al., 2023), neuroprotective agents (Paul and Candelario-Jalil, 2021), and early rehabilitation (Gittler and Davis, 2018; Geng et al., 2022), seem to be related to a better functional outcome.

Recently, increasing evidence indicated that the outcome of IS can also be influenced by gut microbiota (GM). For example, animal model-based studies found that altering the gut microbiome of aged mice after experimental stroke through transplanting youthful microbiota can reverse the poor recovery in aged stroke mice (Spychala et al., 2018; Lee et al., 2020). Another animal study also proved that the cerebral infarct size and post-stroke outcomes were impacted by transplantation of GM, which was associated with the trimethylamine-N-oxide (TMAO) pathway (Zhu et al., 2021). Furthermore, some studies reported the relationship between the composition of GM and the prognosis of human stroke. An observational study reported that *Christensenellaceae*_R-7_group and *norank_f_Ruminococcaceae* were positively correlated with the modified Rankin scale (mRS) at 1 month, which was used to evaluate the stroke outcome, while genus *Enterobacter* was negatively correlated with the mRS (Li et al., 2019). Another case–control study which defined the mRS score of ≥ 3 at 3 months as a poor functional outcome also analyzed the differences of microbiota composition between the outcome groups after stroke, and the results showed that the group with poor outcomes had a higher abundance of *Ruminococcaceae* and *Prevotella* and a lower abundance of *Anaerococcus, Blautia, Dialister, Aerococcaceae, Propionibacterium, Microbacteriaceae*, and *Rothia* compared with the group with good outcomes (Chang et al., 2021). Although the above two research studies confirmed the association between GM and IS functional outcomes, the results did not seem to be entirely consistent, maybe because of the non-uniform functional outcome indicators. In addition, it is uncertain whether these associations are causal, given that the evidence obtained mainly from observational studies may result in potential bias in results due to reverse causation and residual confounding.

Mendelian randomization (MR) is an analytic method based on the summary data of genome-wide association study (GWAS) to establish the causality between exposure and outcome (Sleiman and Grant, 2010; Sekula et al., 2016). Given that genetic variants, such as single-nucleotide polymorphisms (SNPs), are used as proxies for the modifiable environmental factors (exposure) under investigation, the MR method has the advantage of reducing potential bias from confounding factors and reverse causation because of the random assignment of alleles during human gamete formation and the perpetually immutable genotype determined before birth (Sekula et al., 2016). Consequently, with the available GWAS data over the last decade, MR studies have been widely used to analyze the causal association between GM and diseases (Smith and Ebrahim, 2003; Wang et al., 2018; Kurilshikov et al., 2021).

A newly published report has used MR to reveal the causal effect of GM on the risk of IS subtypes (Meng et al., 2023). However, to date, MR studies about the causal inference between GM and IS prognosis remain unavailable. Furthermore, conducting large-scale longitudinal cohort studies or randomized controlled trials (RCTs) currently is unfeasible. In this context, we performed this MR study using the genetic variation associated with GM to assess their possible causal relationship with IS functional outcomes.

## Methods

### Study design

**Figure 1:**
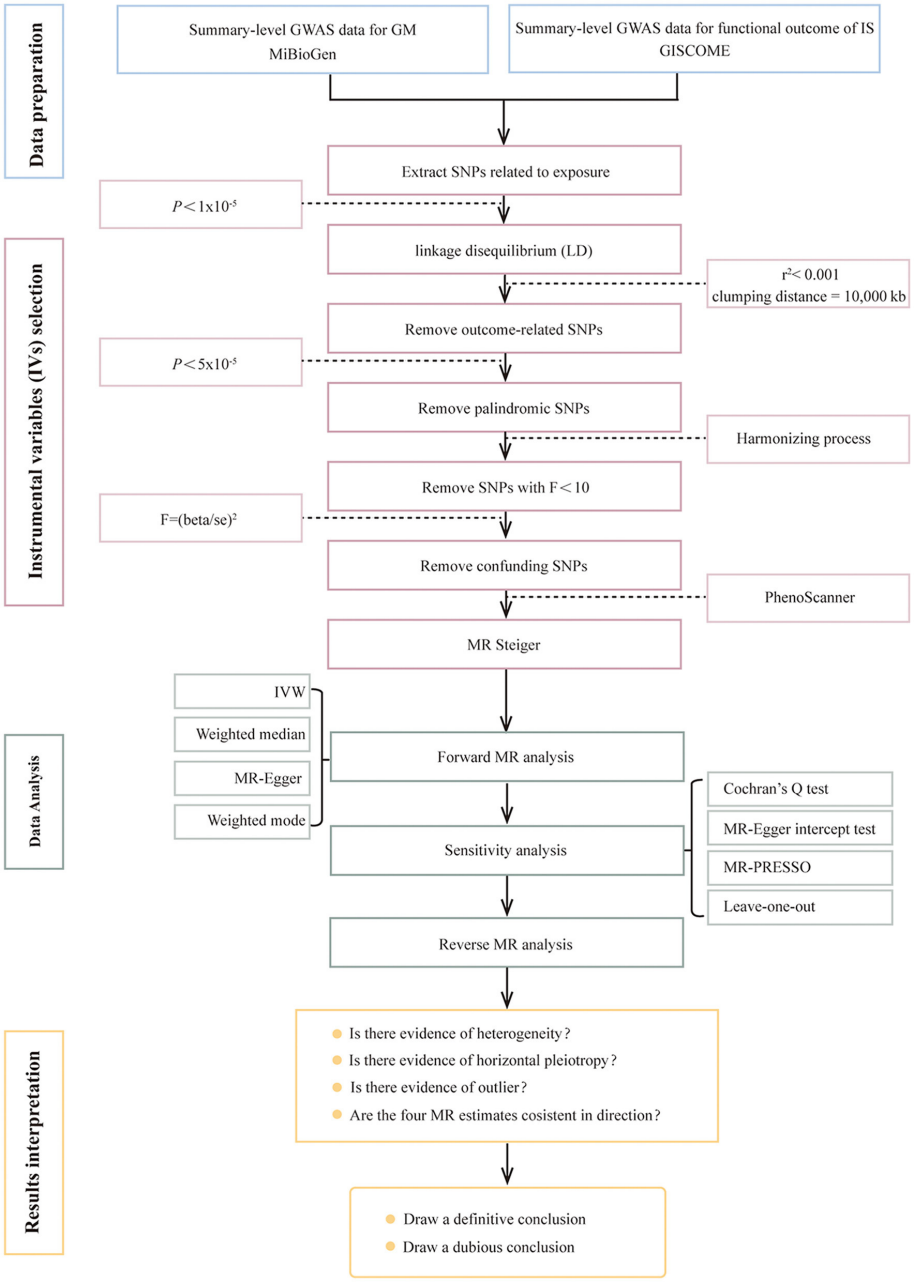
Flowchart of Mendelian randomization analysis. GWAS, genome-wide association study; GM, gut microbiota; IS, ischemic stroke; GISCOME, Genetics of Ischemic Stroke Functional Outcome; IVs, instrumental variables; SNPs, single-nucleotide polymorphisms; LD, linkage disequilibrium; IVW, inverse variance weighted; MR, Mendelian randomization.

This two-sample MR study was designed to explore the causal relationship between genetically predicted GM and functional outcomes after IS. The flowchart of this study is shown in Figure 1. The analysis results are presented in accordance with the Strengthening the Reporting of Observational Studies in Epidemiology-Mendelian Randomization (STROBE-MR) guidelines, which is recommended for this study type (Skrivankova et al., 2021) (Supplementary Table S7).

### Data sources

GWAS data on GM and functional outcomes after IS were acquired from the MiBioGen consortium and the Genetics of Ischemic Stroke Functional Outcome (GISCOME) network, respectively. Notably, populations from both exposure and outcome cohorts were independent and non-overlapping. Moreover, most individuals involved were of European descent, contributing to decreased bias resulting from population stratification. The MiBioGen study was the largest-scale genome-wide meta-analysis of GM to date, identifying a total of 211 taxa from 18,340 individuals (13,266 of European ancestry) of 24 cohorts (Wang et al., 2018). In this study, 196 taxa (comprising of 9 phyla, 16 classes, 20 orders, 32 families, and 119 genera) were ultimately retained and used owing to 15 unknown taxa being excluded. The GISCOME network included 6,021 IS individuals of mainly European ancestry from 12 population-based cohorts and defined mRS score at 3 months after IS as the primary outcome (Soderholm et al., 2019). A dichotomized mRS at 3 months of 0–2 (*n* = 3,741) indicates a better functional outcome and 3–6 (*n* = 2,280) indicates a worse functional outcome. Data on IS prognosis we used in main MR analysis was adjusted for age, sex, ancestry, and baseline NIHSS. Besides, we conducted a comparative analysis without adjusting baseline NIHSS.

### Instrumental variables selection

**Figure 2:**
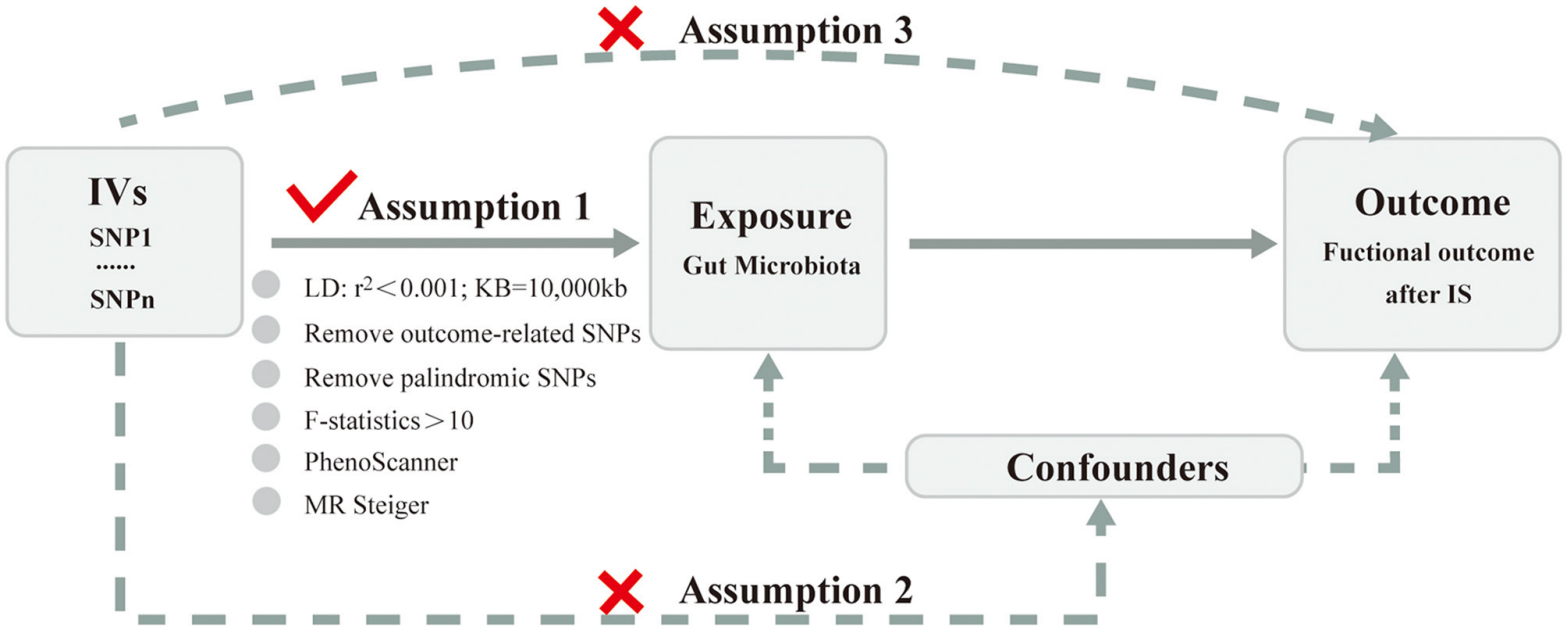
Three key assumptions of Mendelian randomization analysis. IVs, instrumental variables; SNP, single-nucleotide polymorphism; LD, linkage disequilibrium; IS, ischemic stroke.

Choosing genetic variants meeting three key assumptions (Figure 2) as valid IVs is fundamental to obtain a reliable and robust conclusion on causal inference. Valid IVs must be: (i) significantly associated with the exposure (the relevance assumption); (ii) independent of any confounding factors related to the exposure or the outcome (the independence assumption); (iii) associated with the outcome only through the exposure rather than any other ways (the exclusion restriction criteria) (Davies et al., 2018; Carter et al., 2021). Rigorous criteria and steps (Figure 1) were performed as below to obtain the optimal IVs. First, SNPs related to GM were identified as potential IVs under a significance level (*P* < 1 × 10^−5^) (Li C. et al., 2022). Second, SNPs with the lowest *p*-value were eventually retained by performing a linkage disequilibrium (LD) analysis with r^2^ < 0.001 and clumping distance = 10,000 kb based on the data of European samples from the 1,000 Genomes Project. Third, SNPs associated with the outcome under the significance level of *P* < 5 × 10^−5^ were removed after extracting the corresponding information of the selected SNPs from the GWAS outcome data. Fourth, palindromic and ambiguous SNPs were removed during the harmonizing process. Fifth, weak IVs referring to SNPs with F-statistics < 10 were excluded. The F-statistics was calculated using the formula: F = (beta/se)^2^. Finally, SNPs significantly associated (*P* < 5 × 10^−8^) with confounding factors were checked through the PhenoScanner GWAS database and were manually removed. Meanwhile, the MR Steiger test was used to ensure the directional accuracy of the causality for each IV. Only SNPs that were retained through the above screening steps can be finally used for the subsequent MR analysis.

### MR analysis

Four different methods were used for this bidirectional MR study to explore the causal relationship between GM and IS functional outcomes. Inverse variance weighted (IVW) was conducted as the primary analysis approach, complemented by the MR-Egger, weighted median, and weighted mode methods (Long et al., 2023). IVW can provide unbiased causal results in the case of the balanced horizontal pleiotropy or no horizontal pleiotropy (Hemani et al., 2018). Under the assumption that instrument strength is independent of direct effect (InSIDE), MR-Egger regression could provide evidence of no horizontal pleiotropy and a consistent result with IVW if the intercept equals zero (Bowden et al., 2015). The efficiency of weighted median is similar to IVW if up to 50% of the weights is from valid IVs (Bowden et al., 2016). The weighted mode method is proved to have fewer biases than MR-Egger regression if InSIDE assumption is falsified (Li P. et al., 2022). As a whole, if the results of the above methods are inconsistent, the results of IVW will be given priority.

To address multiple comparisons (196 exposures), *p*-values of the IVW method have been adjusted by false discovery rate (FDR) correction. *P*_FDR_ < 0.05 was considered to indicate a significant association (Xu et al., 2021; Gu et al., 2023).

### Sensitivity analysis

Sensitivity analysis for potential heterogeneity and horizontal pleiotropy have also been performed to detect the robustness of causal inference results. The Cochran's Q test with an insignificant *P* value (*P* > 0.05) was defined as having no heterogeneity. The MR-Egger intercept test with an insignificant *P* value (*P* > 0.05) indicated no horizontal pleiotropy. Furthermore, the MR-PRESSO test was performed to eliminate the effect of horizontal pleiotropy by re-analyzing it after removing pleiotropic SNPs. The leave-one-out analysis was applied to rule out potential pleiotropy driven by a single SNP by excluding one instrumental SNP each time and repeating the IVW analysis.

All statistical analyses, including the MR analysis and the sensitivity analysis, were performed using TwoSampleMR and MRPRESSO packages in R (version 4.2.1). In addition, ggplot2 package in R was used for data visualization.

## Results

### IV selection

We first obtained 196 taxa at the phylum, class, order, family, and genus levels after excluding 15 unknown taxa (Supplementary Table S1). Subsequently, several screening steps mentioned above were implemented. Confounding factors, such as smoking (Zhang et al., 2022), migraine (Wang et al., 2023), frailty (Cai et al., 2023), depression (Gill et al., 2019), diabetes (Lau et al., 2019), body mass index (BMI), and insomnia (Zhang et al., 2023), were determined by reviewing the literature. We manually removed 35 SNPs, and a total of 2038 SNPs from 196 taxa were eventually chosen as IVs (Supplementary Table S2).

### Causal effects of GM on functional outcomes after IS

**Figure 3:**
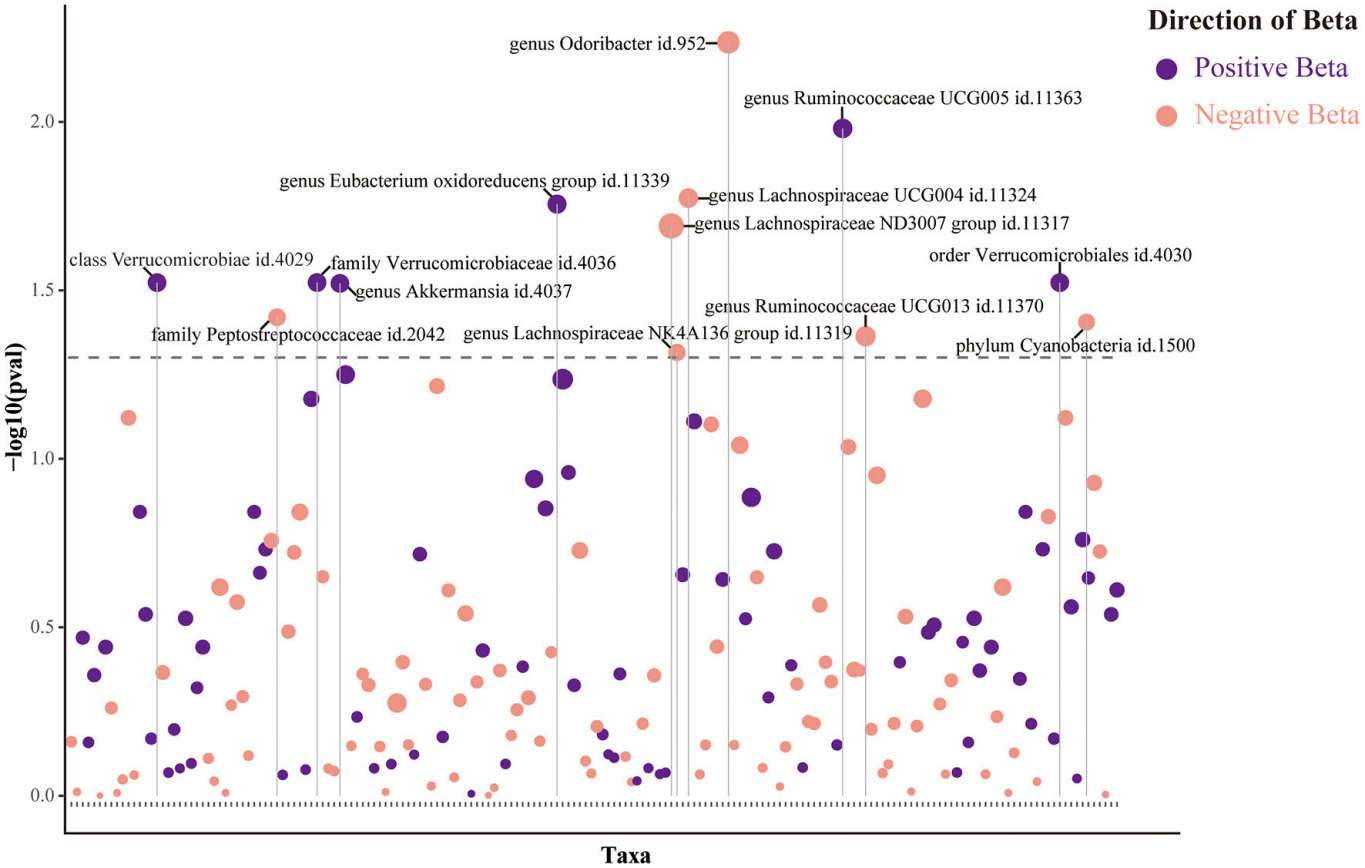
Lollipop plot was constructed to illustrate the outcomes of the IVW analysis concerning the impact of 196 gut microbiota (GM) taxa on functional outcomes after ischemic stroke (IS). In this plot, positive beta values are represented in purple, while negative beta values are represented in pink. Dashed lines positioned above the plot indicate *p*-values below the 0.05 threshold. Taxa that achieved statistical significance are explicitly labeled in the plot.

**Figure 4:**
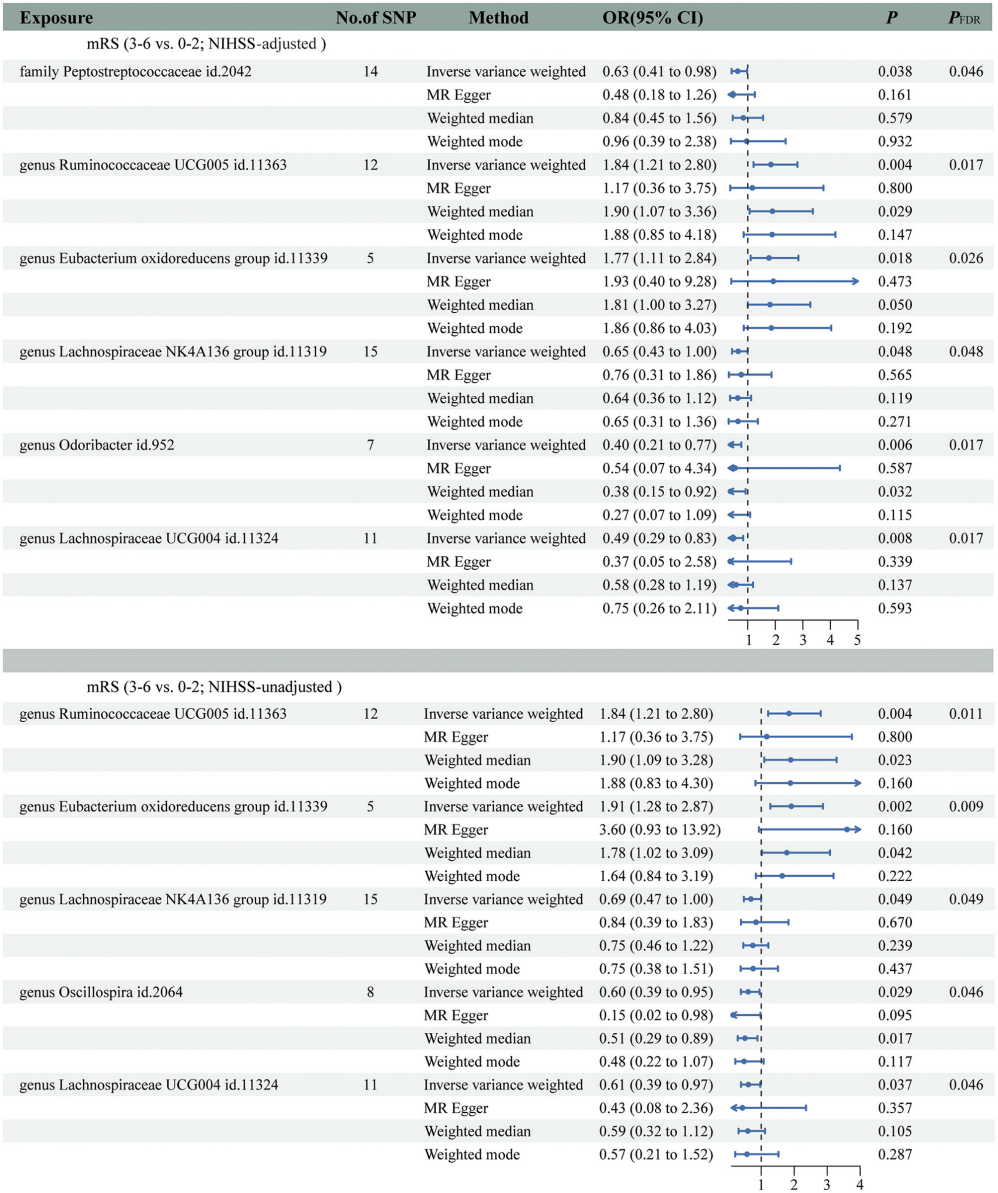
Forest plot was used to present the results of four analyses on the genetic associations between gut microbiota (GM) and functional outcomes after ischemic stroke (IS).

**Figure 5:**
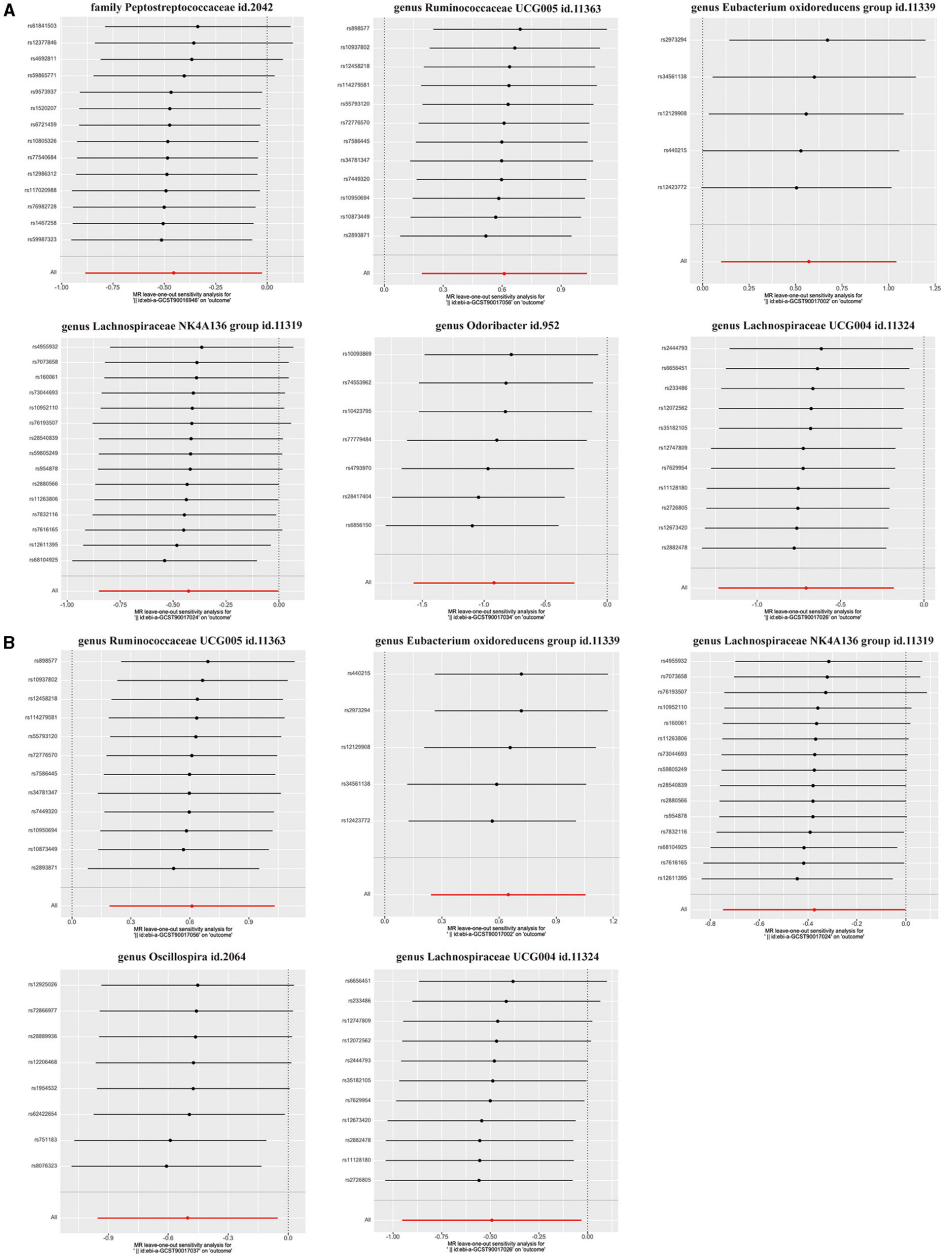
Leave-one-out analysis for **(A)** six GM taxa in the main analysis, **(B)** five GM taxa in the comparative analysis.

The main IVW results of 196 GM taxa in the forward MR analysis were shown in the lollipop plot in Figure 3. A total of 13 taxa which have the possibility of causal associations with the functional outcome after IS were initially selected. We further excluded class *Verrucomicrobiae*, family *Verrucomicrobiaceae*, genus *Akkermansia*, genus *Lachnospiraceae ND3007* group, genus *Ruminococcaceae UCG013*, order *Verrucomicrobiales*, and phylum *Cyanobacteria* from the analysis results because of the inconsistent direction of effect estimates produced by the four MR methods (Supplementary Table S3). Finally, a total of six significant GM taxa were obtained. The Forest plot of four analyses is shown in Figure 4. Based on the results of the IVW analysis, genus *Ruminococcaceae UCG005* (odds ratio [OR] = 1.842, 95% confidence interval [CI] 1.210–2.804, *P* = 0.004, *P*_FDR_ = 0.017) and the genus *Eubacterium oxidoreducens* group (OR = 1.771, 95% CI 1.105–2.837, *P* = 0.018, *P*_FDR_ = 0.026) were demonstrated to have a positive correlation with worse functional outcomes after IS. Family *Peptostreptococcaceae* (OR = 0.635, 95% CI 0.413–0.975, *P* = 0.038, *P*_FDR_ = 0.046), the genus *Lachnospiraceae NK4A136* group (OR = 0.653, 95% CI 0.427–0.997, *P* = 0.048, *P*_FDR_ = 0.048), genus *Lachnospiraceae UCG004* (OR = 0.493, 95% CI 0.292–0.834, *P* = 0.008, *P*_FDR_ = 0.017), and genus *Odoribacter* (OR = 0.399, 95% CI 0.208–0.766, *P* = 0.006, *P*_FDR_ = 0.017) were negatively correlated with worse functional outcomes after IS. According to the MR estimates of the weighted median, genus *Odoribacter* (OR = 0.377, 95% CI, 0.154–0.920, *P* = 0.032) was considered as protective factors for functional outcomes after IS, whereas genus *Ruminococcaceae UCG005* (OR = 1.895, 95% CI 1.068–3.362, *P* = 0.029) and the genus *Eubacterium oxidoreducens* group (OR = 1.809, 95% CI 1.068–3.270, *P* = 0.0499) were considered as risk factors (Figure 4). The results of the sensitivity analysis for six significant taxa show no evidence of horizontal pleiotropy and heterogeneity (Supplementary Table S4). The MR-PRESSO global test (*P* > 0.05) indicated no outliers in the results. Furthermore, the leave-one-out analysis did not provide any evidence that one single SNP was responsible for the inferred causal relationship between GM and functional outcomes after IS (Figure 5). The results of the above analysis confirmed the accuracy and robustness of causal inference of genetically predicted GM and functional outcomes after IS.

In addition, the positive results of the analysis without adjustment for the baseline NIHSS were used as a comparison. Notably, we obtained five significant taxa (Figure 4), four of which had similar results to the main analysis, and no evidence of pleiotropy, heterogeneity or outliers were found (Supplementary Table S4). The results of leave-one-out analysis from the comparative analysis are presented in Figure 5. Similar to the main IVW analysis, genus *Ruminococcaceae UCG005* (OR = 1.842, 95% CI 1.210–2.804, *P* = 0.004, *P*_FDR_ = 0.011) and the genus *Eubacterium oxidoreducens* group (OR = 1.913, 95% CI 1.275–2.871, *P* = 0.002, *P*_FDR_ = 0.009) showed a positive correlation with the worse prognosis after IS, whereas genus *Lachnospiraceae NK4A136 group* (OR = 0.688, 95% CI 0.473–0.999, *P* = 0.049, *P*_FDR_ = 0.049) and genus *Lachnospiraceae UCG004* (OR = 0.612, 95% CI 0.386–0.970, *P* = 0.037, *P*_FDR_ = 0.046) showed a negative correlation with worse prognosis after IS. Genus *Oscillospira* (OR = 0.605, 95% CI 0.385–0.949, *P* = 0.029, *P*_FDR_ = 0.046) also showed a negative correlation with the poor outcome in the comparative analysis, which is different from Family *Peptostreptococcaceae* and genus *Odoribacter* in the main analysis. In terms of risk factors for functional outcomes after IS, the weighted median method produced results similar to the main analysis, further suggesting the confidence of genus *Ruminococcaceae UCG005* (OR = 1.895, 95% CI 1.094–3.284, *P* = 0.023) and genus *Eubacterium oxidoreducens* group (OR = 1.776, 95% CI 1.022–3.086, *P* = 0.042) as risk factors. However, for protective factors, the weighted median method yielded a different result than the main analysis for the genus *Oscillospira* (OR = 0.506, 95% CI, 0.289–0.886, *P* = 0.017).

### Causal effects of functional outcomes after IS on GM

We also performed a reverse MR analysis on seven positive taxa that were identified to be causally related to functional outcomes after IS in the forward MR analysis to explore the direction of causation. The reverse MR analysis showed no suggestive causal association between prognosis of IS and GM (Supplementary Table S5). Additionally, the results of the sensitivity analysis did not provide any evidence of horizontal pleiotropy and heterogeneity (Supplementary Table S6).

## Discussion

To our knowledge, this is the first MR analysis, overcoming environmental confounding and reverse causation, to investigate the causal effect of genetically determined gut microbiota on functional outcomes after ischemic stroke. Evidence from this study supported a causal association between the abundance of specific bacterial traits and the prognosis of IS. However, positive findings in this study were mainly at the genus level, and no causal associations between GM and IS prognosis at the level of the phylum, class, and order were found. Strikingly, genus *Ruminococcaceae UCG005* and the genus *Eubacterium oxidoreducens* group showed significantly negative effects on stroke prognosis, while genus *Lachnospiraceae NK4A136 group* and genus *Lachnospiraceae UCG004* showed protective effects against stroke prognosis in both main and comparative IVW analysis. Additionally, although the results of the main and comparative weighted median analyses were inconsistent in terms of protection factors, genus *Ruminococcaceae UCG005* and genus *Eubacterium oxidoreducens* group as risk factors for poor prognosis of IS were indisputable. We also performed a reverse MR analysis of these positive taxa, and the results did not support a causal effect of post-stroke prognosis on these taxa. These findings may provide important implications for the discovery of novel biomarkers in future IS experiments and provide prevention and therapeutic strategies targeting dysbiosis of specific GM taxa.

Previous research found that *Ruminococcaceae UCG005* increased in severe stroke patients who usually have a worse stroke outcome (Li et al., 2019). This is consistent with our research. A network analysis suggested that *Ruminococcaceae UCG005* was one of the genera driving the progress of the type 2 diabetes in a Mexican cohort (Esquivel-Hernandez et al., 2023). An animal study reported a significantly negative correlation of *Ruminococcaceae UCG005* with high-density lipoprotein cholesterol (HDL-C) and a positive correlation with body weight (Qin et al., 2022). It is well known that abnormal glucose and lipid metabolism are the dominant causes of cerebrovascular diseases. Genus *Ruminococcaceae UCG005* may influence the stroke prognosis by the pathway of glucose and lipid metabolism. Furthermore, ample evidence shows that imbalance in GM contributes to the neuroinflammation and worse stroke outcomes (Huang et al., 2023; Park et al., 2023). An animal-based study found that maternal sleep deprivation (MSD) caused high expression of pro-inflammatory cytokines in offspring rats Moreover, pro-inflammatory cytokines were positively associated with *Ruminococcaceae UCG005* (Yao et al., 2022). Therefore, it is speculated that *Ruminococcaceae UCG005* may also be involved in the inflammatory response after stroke, thereby adversely affecting the prognosis of stroke. These results may support the conclusion of *Ruminococcaceae UCG005* as a risk factor for stroke prognosis.

For the *Eubacterium oxidoreducens* group, we failed to find some direct evidence of its role in stroke prognosis from existing research. Research on the role of the *Eubacterium oxidoreducens* group in other diseases is also rare. However, a previous study reported a significantly elevated relative abundance of E*ubacterium oxidoreducens* group which was positively correlated with the levels of serum and fecal lipopolysaccharide (LPS) in high-fat diet (HFD)-fed mice (Zhang X. Y. et al., 2020). Increased LPS contents could impair the intestinal epithelial barrier (IEB) (Guo et al., 2015) and blood–brain barrier (BBB) (Peng et al., 2021). Recent studies have revealed that gut barrier integrity and BBB are involved in the influence of GM on IS (Gwak and Chang, 2021; Zeng et al., 2023). A new study found that intraperitoneal injection of LPS after stroke exhibited intestinal morphology damage, decreased expression of tight-junction proteins associated with the BBB, and more neuronal loss, and these changes were consistent with stroke mice transplanted with gut microbiota associated with post-stroke cognitive impairment (Wang et al., 2022). Therefore, we speculate that the *Eubacterium oxidoreducens* group, as a risk factor, may have adverse effects on stroke prognosis by affecting intestinal epithelial integrity and blood–brain barrier.

The results of another MR study evaluating the causal effect of GM on cardioembolic IS support the protective effect of the genus *Lachnospiraceae NK4A136* group on IS prognosis in this MR analysis (Dai et al., 2024). A previous study reported a significant negative association of the genus L*achnospiraceae NK4A136* group with intestinal permeability and the plasma LPS level (Ma et al., 2020), which means that genus *Lachnospiraceae NK4A136* group is beneficial for protecting the intestinal barrier. The intestinal barrier acts as the first barrier to prevent harmful substances from penetrating the intestinal mucosa and damaging other tissues of the body. The disruption of IEB after stroke contributes to the microbial translocation which will increase the risk of post-stroke infections (Zhao et al., 2023). Therefore, the protective effect of the genus *Lachnospiraceae NK4A136* group on IEB may be helpful in IS prognosis.

High abundance of genus *Lachnospiraceae UCG004* was also identified useful for IS prognosis. As probiotics in the body, it is beneficial for reducing obesity (Xu et al., 2024). Evidence from a clinical study also showed a negative correlation between *Lachnospiraceae UCG004* and cardiovascular disease risk factors (Tindall et al., 2020). Another case–control study found a low abundance of *Lachnospiraceae UCG004* in lacunar cerebral infarction patients compared with healthy controls (Ma et al., 2023). It is worth noting that both the genus L*achnospiraceae NK4A136* group and *Lachnospiraceae UCG004* belong to family *Lachnospiraceae* which is recognized as short chain fatty acid (SCFA)-producer. In most studies, increased inflammation response and decreased SCFAs could be observed in stroke individuals and were significantly related to poor IS outcomes (Spychala et al., 2018; Tan et al., 2021). Animal-based studies proved that transplantation of SCFAs-rich gut microbiota or SCFA supplementation in drinking water can effectively promote the recovery following ischemic stroke (Chen et al., 2019; Lee et al., 2020; Sadler et al., 2020). Studies showed that SCFAs participate in the regulatory process of inflammatory responses, including promoting the anti-inflammatory cytokines and suppressing the pro-inflammatory cytokines (Maslowski et al., 2009; Vinolo et al., 2011). This regulatory effect of SCFAs on inflammation may be one of the mechanisms by which GM affects the prognosis of stroke (Iadecola and Anrather, 2011; Chamorro et al., 2012).

The underlying mechanisms involved in the influence of GM on outcomes following IS are multifaceted. In addition to the above-mentioned content, neurotransmitters and TMAO pathway (Zhu et al., 2021), among others are involved in the regulation of GM on stroke. In conclusion, more research is needed to confirm whether and how the four taxa identified in this study are involved in these mechanisms.

To date, most studies evaluating the role of gut microbiome in stroke have focused on bacteria due to the overwhelming abundance of bacteria. Research on viruses, fungi, and archaea is scarce. However, these non-bacteria gut microbes play an important role in human health and diseases (Goralska et al., 2018; Mukhopadhya et al., 2019; Coker, 2022; Ezzatpour et al., 2023). There is a growing body of evidence that suggests non-bacteria gut microbes are associated with neurological diseases, including stroke (Forbes et al., 2018). A new study found that fecal viral taxa was altered significantly after stroke and dissimilar phage protein networks in mice (Chelluboina et al., 2022). Phages are regarded as the most identified human gut virus components with significant roles. Phages can infect diverse bacterial phyla in the gut, such as Firmicutes, Bacteroidetes, Proteobacteria, and Actinobacteria (Mirzaei and Maurice, 2017). When healthy people took phage dietary supplements orally, there was an increase in the butyrate-producing genus, *Eubacterium* (Febvre et al., 2019). In addition to the viruses, some fungi housed in the gastrointestinal tract are considered to be pathogenic and can destroy CNS astrocytes, leading to BBB disruption and central infection. Colonizing the gut by archaea has also been demonstrated to decrease levels of trimethylamine (Forbes et al., 2018), a compound produced by intestinal bacteria that is linked to an increased risk of atherosclerosis as well as cardiovascular and cerebrovascular diseases. Hence, the role of non-bacterial microorganisms in stroke is also one of the important directions of future stroke research. Unfortunately, there is no GWAS data for non-bacterial microorganisms, and the causal relationship between stroke and stroke prognosis cannot be further explored in this study. Nevertheless, we are eagerly looking forward to the improvement of the GWAS data that will enable us to delve deeper into this fascinating area of research in the future.

The MR analysis we designed is unlikely to be biased because of confounding factors and reverse causality. We also drew a relatively consistent and reliable conclusion from the data obtained from two sets of outcomes which were with or without adjustment for the baseline NIHSS. Although we have tried our best to make the conclusions accurate and robust, there are still some limiting factors that need to be considered in our research. First, the study's limitation in not being able to mine data below the genus level indeed restricts the depth of understanding of the potential links between the gut microbiota and outcomes of ischemic stroke. Second, a test about the associations between GM and post-stroke outcomes for different stroke subtypes was not performed because of the lack of available GWAS data in the GISCOME database. Different types, localization of stroke have a decisive influence on recovery in survivors (Biffi et al., 2015; Zhang K. et al., 2020), which may contribute to some bias in our results. Third, we did not adequately control for age and sex among the patients included in the study. Thus, age and sex need to be taken into account in further investigations on the relationship between GM and post-stroke outcomes because the formation and shaping of the GM are easily influenced by sex and lifespan (Snigdha et al., 2022). Fourth, a previous MR analysis has indicated that genetically determined GM was associated with the onset of ischemic stroke (Meng et al., 2023), which made the collider bias a non-negligible issue in this MR investigation of IS prognosis (Paternoster et al., 2017). Fifth, a small sample size of subjects (*n* = 6,021) included in this study were only of European ancestry, which may restrict the generalizability of our findings to other populations. Thus, future studies with a larger sample size of other ancestries are needed to explore the associations between GM and ischemic stroke outcomes.

## Conclusion

In summary, this MR analysis demonstrated that genetically predicted gut microbiota is causally associated with worse post-stroke outcomes. Our results suggest that interventions addressing particular GM taxa, such as *Lachnospiraceae NK4A136, Lachnospiraceae UCG004, Ruminococcaceae UCG005*, and *Eubacterium oxidoreducens* groups, may provide new opportunities to improve recovery after ischemic stroke.

## Data availability statement

The original contributions presented in the study are included in the article/Supplementary material, further inquiries can be directed to the corresponding authors.

## Author contributions

AZ: Conceptualization, Writing – review & editing. PL: Software, Writing – review & editing. YC: Software, Writing – review & editing. XW: Data curation, Writing – review & editing. JZ: Data curation, Writing – review & editing. LL: Methodology, Writing – review & editing. TZ: Conceptualization, Writing – original draft. JY: Funding acquisition, Writing – original draft.
